# Supplementary material for: Physical activity and aerobic fitness in relation to local and interhemispheric functional connectivity in adolescents’ brains
Source: Brain Behav. 2020 Dec 24;11(2):e01941. doi: 10.1002/brb3.1941 (PMC7882164; doi:10.1002/brb3.1941)
Supplement: Supplementary file 1 [file BRB3-11-e01941-s001.docx]

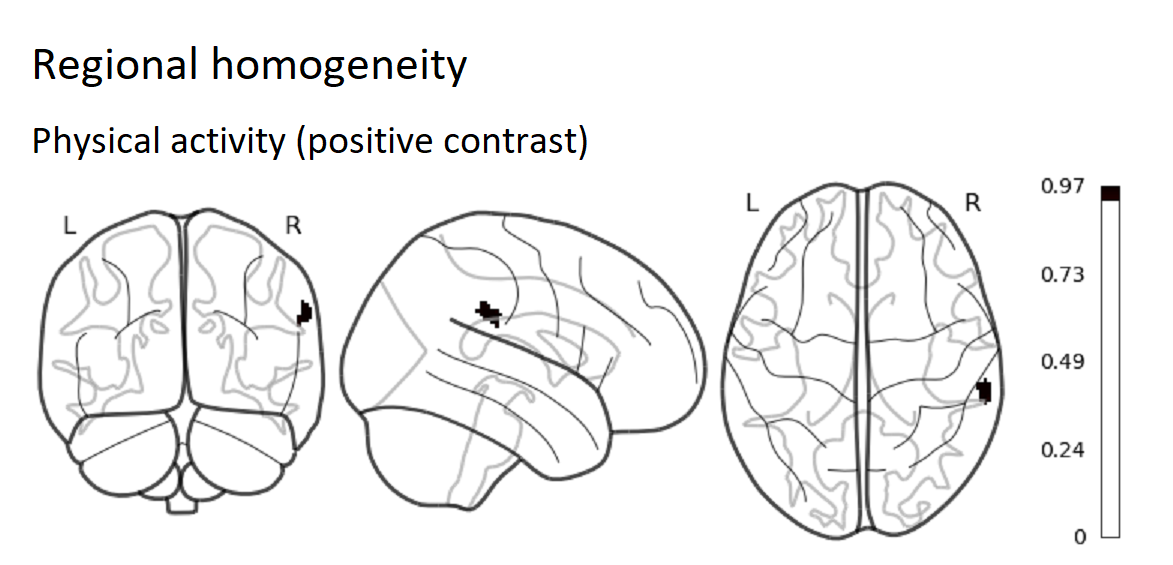


**Supplementary Figure 1**

Group-level 1-p-value map regarding positive correlation between moderate-to-vigorous physical activity and ReHo (p<0.05, corrected for TFCE and family-wise error rate), when taking into account mean frame-wise displacement.
